# Neurological manifestations of severe acute respiratory syndrome coronavirus 2—a controversy ‘gone viral’

**DOI:** 10.1093/braincomms/fcaa149

**Published:** 2020-09-17

**Authors:** Moritz Förster, Vivien Weyers, Patrick Küry, Michael Barnett, Hans-Peter Hartung, David Kremer

**Affiliations:** Department of Neurology, Medical Faculty, Heinrich-Heine-University, 40225 Düsseldorf, Germany; Department of Neurology, Medical Faculty, Heinrich-Heine-University, 40225 Düsseldorf, Germany; Department of Neurology, Medical Faculty, Heinrich-Heine-University, 40225 Düsseldorf, Germany; Department of Neurology, Royal Prince Alfred Hospital, University of Sydney, Sydney, NSW, Australia; Brain and Mind Centre, University of Sydney, Sydney, Australia; Department of Neurology, Medical Faculty, Heinrich-Heine-University, 40225 Düsseldorf, Germany; Center of Neurology and Neuropsychiatry, LVR Klinikum, Medical Faculty, Heinrich-Heine-University, 40629 Düsseldorf, Germany; Department of Neurology, Medical Faculty, Heinrich-Heine-University, 40225 Düsseldorf, Germany

**Keywords:** COVID-19, SARS-CoV-2, neurological manifestations

## Abstract

Severe acute respiratory syndrome coronavirus 2 first appeared in December 2019 in Wuhan, China, and developed into a worldwide pandemic within the following 3 months causing severe bilateral pneumonia (coronavirus disease 2019) with in part fatal outcomes. After first experiences and tentative strategies to face this new disease, several cases were published describing severe acute respiratory syndrome coronavirus 2 infection related to the onset of neurological complaints and diseases such as, for instance, anosmia, stroke or meningoencephalitis. Of note, there is still a controversy about whether or not there is a causative relation between severe acute respiratory syndrome coronavirus 2 and these neurological conditions. Other concerns, however, seem to be relevant as well. This includes not only the reluctance of patients with acute neurological complaints to report to the emergency department for fear of contracting severe acute respiratory syndrome coronavirus 2 but also the ethical and practical implications for neurology patients in everyday clinical routine. This paper aims to provide an overview of the currently available evidence for the occurrence of severe acute respiratory syndrome coronavirus 2 in the central and peripheral nervous system and the neurological diseases potentially involving this virus.

## Teaser

Severe acute respiratory syndrome coronavirus 2 may be involved in or contribute to a plethora of neurological diseases. However, this hypothesis remains controversial. We aim to provide an overview of the currently available evidence for the occurrence of severe acute respiratory syndrome coronavirus 2 in the human nervous system and the potential sequelae.

## Graphical Abstract

**Graphical Abstract:**
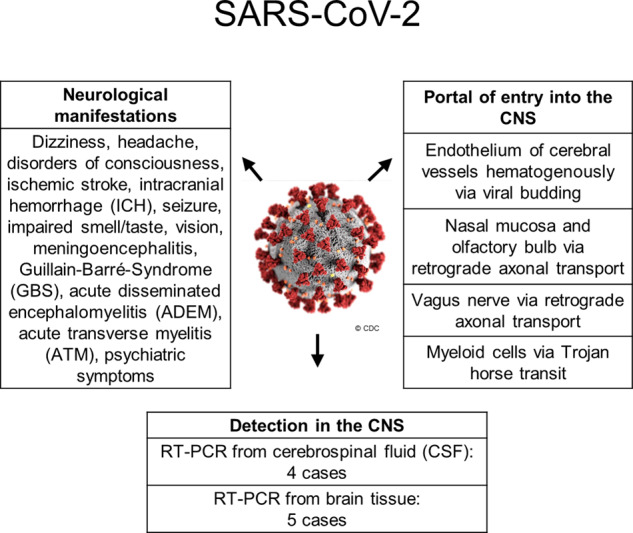

## Introduction

Severe acute respiratory syndrome coronavirus 2 (SARS-CoV-2), a novel β-corona single-stranded RNA virus, first appeared in Wuhan, China, in December 2019 and developed into a worldwide pandemic within the following 3 months. This makes it the third coronavirus epidemic after the outbreak of severe acute respiratory syndrome coronavirus (SARS-CoV) in 2003 and middle east respiratory syndrome coronavirus in 2012. Beyond its enormous multi-faceted medical aspects, the pandemic also challenges us to re-think daily hospital routines ranging from the implementation of telephone and video consultations for patients to the organization of interdisciplinary e-conferences (Grossman *et al.*, 2020; McArthur, 2020). Similar to other human coronaviruses, SARS-CoV-2 primarily targets the upper and lower respiratory system and was first isolated from cases of bilateral pneumonia termed coronavirus disease 2019 (COVID-19; Lau and Peiris, 2005; Hirano and Murakami, 2020; Rockx *et al.*, 2020; Wang *et al.*, 2020*a*; Zhou *et al.*, 2020). Apart from this, numerous studies have been published that describe nervous system involvement in SARS-CoV-2-positive patients (Table 1). Of the four SARS-CoV-2 structural proteins spike, membrane, envelope and RNA-containing nucleocapsid, the spike protein is responsible for viral pathogenicity by binding to the human angiotensin converting enzyme 2 (ACE2) receptor. Of note, SARS-CoV-2 binds to ACE2 receptor with a 10–20-fold higher affinity than SARS-CoV. ACE2 receptor is, *inter alia*, expressed on the lung epithelium (Hirano and Murakami, 2020) where its activation ultimately results in an infiltration of lung alveoli by activated T lymphocytes, natural killer cells and activated monocytes/macrophages. Moreover, it causes a deleterious cytokine release syndrome (i.e. cytokine storm). In the brain, ACE2 receptor can be found in the piriform cortex (Doobay *et al.*, 2007), the brainstem (Lenkei *et al.*, 1996; Lin *et al.*, 2008) and cardiovascular regulatory areas (Doobay *et al.*, 2007; Yamazato *et al.*, 2007; Feng *et al.*, 2008) such as the subfornical organ, the paraventricular nucleus, the nucleus of the tractus solitarius and the rostral ventrolateral medulla. On the cellular level, it was found on neurons (Doobay *et al.*, 2007; Xiao *et al.*, 2013; Mukerjee *et al.*, 2019; Chen et al., 2020*b*; Zhu et al., 2020), glial cells (Gallagher *et al.*, 2006; Gowrisankar and Clark, 2016; Chen et al., 2020*b*; Zhu *et al.*, 2020) and non-neural olfactory epithelial cells [i.e. horizontal basal cells and Bowman’s gland cells (Brann *et al.*, 2020)]. Interestingly, among CNS cells, oligodendrocytes seem to be of particular interest as they express not only ACE2 but also the transmembrane protease, serine 2, an important SARS-CoV-2 co-receptor (Needham *et al.*, 2020). Epidemiologically, it remains currently unclear how many COVID-19 patients exhibit neurological complications with numbers ranging from a few per cent to up to 80% (Helms *et al.*, 2020*a*; Mao *et al.*, 2020). However, whether or not these complications are based on direct effects of the virus on the central nervous system CNS or the peripheral nervous system still needs to be clarified.

**Table 1:** Nervous system manifestation in SARS-CoV-2-positive patients

Authors	Study type	Definition COVID-19 positive	SARS-CoV2-positive patients included	Age (years)	Female/male/ diverse in %	Dizziness (%)	Headache (%)	Disorders of consciousness (%)	Ischaemic stroke (%)	ICB (%)	Seizure (%)	Patients with impaired		
												Smell (%)	Taste (%)	Vision (%)
Romero-Sánchez *et al.* (2020)	Retrospective observational two-centre (ALBACOVID registry)	Throat swab RT-PCR or immunoglobulin G/immunoglobulin M antibodies against SARS-CoV-2 in a blood test	841	Mean 66.4 (±SD 15.0)	56.2/43.8/0	51 (6.1)	119 (14.1)	165 (19.6)	11 (1.3)	3 (0.3)	6 (0.7)	41 (4.9)	52 (6.2)	n.a.
Lodigiani *et al.* (2020)	Retrospective observational single-centre	Laboratory-proven COVID-19	388	Median 66 (IQR 55.0–75.0)	32.0/68.0	n.a.	n.a.	n.a.	9/362 closed cases (2.5)	n.a.	n.a.	n.a.	n.a.	n.a.
Chen *et al*. (2020d)	Retrospective case study, single- centre	Throat swab RT-PCR	274	Median 62.0 (IQR 44.0–70.0)	37.6/62.4/0	21 (7.6)	31 (11.3)	26 (9.5)	n.a.	n.a.	n.a.	n.a.	n.a.	n.a.
Li *et al.*, (2020*a*, )	Retrospective observational single-centre	Confirmed COVID-19	221	n.a.	n.a.	n.a.	n.a.	n.a.	11 (5.0)	1 (0.5)	n.a.	n.a.	n.a.	n.a.
Mao *et al.* (2020)	Retrospective observational multicentre	Throat swab RT-PCR	214	Mean 52.7 (±SD 15.5)	59.3/40.7/0	36 (16.8)	28 (13.1)	16 (7.5)	5 (2.3)	1 (0.5)	1 (0.5)	12 (5.6)	11 (5.1)	3 (1.4)
Chen *et al.* (2020*a*, *b*, *c*)	Retrospective observational single-centre	Throat swab RT-PCR	99	Mean 55.5 (±SD 13.1)	32.3/67.7/0	n.a.	8 (8.1)	9 (8.1)	n.a.	n.a.	n.a.	n.a.	n.a.	n.a.
Giacomelli *et al*. (2020)	Questionnaire-based, patient-reported, cross-sectional, single-centre	n.a.	59	Median 60 (IQR 50–74)	32.2/67.8	n.a.	2 (3.4)	n.a.	n.a.	n.a.	n.a.	14 (23.7)	17 (28.8)	n.a.
Yan *et al*. (2020)	Questionnaire-based, patient-reported, cross-sectional, single-centre	Throat swab RT-PCR	59	n.a.	49.2/49.2/1.7	n.a.	39 (66.1)	n.a.	n.a.	n.a.	n.a.	40 (67.8)	42 (71.2)	n.a.
Yang *et al*. (2020)	Retrospective observational single-centre	Throat swab RT-PCR	52	Mean 51.9 (±SD 12.9)	32.6/67.3/0	n.a.	3 (5.8)	n.a.	n.a.	n.a.	n.a.	n.a.	n.a.	n.a.
Oxley *et al.* (2020)	Case series	Throat swab RT-PCR	5	Median 39 (IQR 35.0–46.5)	20.0/80.0	n.a.	1 (20.0)	4 (80.0)	5 (100.0)	0 (0.0)	0 (0.0)	n.a.	n.a.	n.a.

The table shows a selection of observational and case studies that describe neurological manifestations in SARS-CoV-2-positive patients. The study listing is based on the number of included patients. A selection of frequently mentioned neurological complaints in the context of a SARS-CoV-2 infection was made. ICB = intracranial haemorrhage; IQR = interquartile range; n.a. = data not given or not available to the authors; SD = standard deviation.

On the one hand, COVID-19-associated neurological symptoms could be simply based on sepsis or multiorgan failure (e.g. lung dysfunction with subsequent hypoxia), or the above-mentioned cytokine storm featuring production of pro-inflammatory cytokines such as interleukin (IL)-1β, IL-2, IL-6, IL-7, IL-8, tumour necrosis factor-α, C-X-C motif chemokine 10 and chemokine ligand 2 (Helms *et al.*, 2020*a*; Huang *et al.*, 2020*a*; Mehta *et al.*, 2020; Wan et al., 2020). Particularly, tumour necrosis factor-α might destabilize the blood–brain-barrier rendering the CNS vulnerable (Kim *et al.*, 1992). As a consequence, CNS-resident cells such as microglia or astrocytes could be driven to attack other cells in the brain and spinal cord leading to parenchymal injury. On the other hand, a conceivable mechanism of direct SARS-CoV-2-mediated CNS damage could consist in an invasion of the virus into the CNS (Table 2). In rodent animal models, mice were exposed to intranasal injections with the SARS-CoV-2-related human β-*coronavirus* OC43. Via the nasal mucosa and olfactory bulb, the virus was found to use retrograde axonal transport in order to travel to the brain and brainstem where it might contribute to dysregulated breathing or to compromised pulmonary and cardiac functions (Butler *et al.*, 2006). This portal of entry could also explain the loss of smell and taste in the early stages of the disease or oedematous changes in the olfactory bulb (Laurendon *et al.*, 2020; Mao *et al.*, 2020; Meng *et al.*, 2020; Vaira *et al.*, 2020). Of note and interestingly, it has been known for a long time that infection of mice with neurotropic strains of the coronavirus mouse hepatitis virus leads to CNS demyelination mimicking multiple sclerosis (Haring and Perlman, 2001). Moreover, SARS-CoV-2 might retrogradely travel from the lung or the intestines to the CNS via the vagus nerve (Li *et al.*, 2020*b*; Machado and Gutierrez, 2020). In addition, it could be shown that human coronaviruses and especially SARS-CoV are able to infect and activate myeloid cells such as monocytes, which may then invade the CNS haematogenously via Trojan horse transit (Law *et al.*, 2005; Yilla *et al.*, 2005; Chen et al., 2020*c*; Moore and June, 2020). Finally, it is conceivable that in the context of viremia blood-borne SARS-CoV-2 travels to the CNS where it infects the endothelium and might then reach the brain via viral budding (Varga *et al.*, 2020). In summary, each of the above-described mechanisms could help to explain the occurrence of neurological symptoms and diseases in SARS-CoV-2-positive patients.

**Table 2:** Potential portals of entry for SARS-CoV-2 into the CNS

Portal of entry into the CNS	Mode of action	Authors
Endothelium of cerebral vessels	Haematogenously via viral budding	Paniz-Mondolfi *et al.* (2020); Varga *et al.* (2020)
Nasal mucosa and olfactory bulb	Via retrograde axonal transport	Laurendon *et al.* (2020); Meng *et al.* (2020)
Vagus nerve	Via retrograde axonal transport	Li *et al.* (2020*b*); Machado and Gutierrez (2020)
Myeloid cells	Haematogenously via Trojan horse transit	Chen *et al.* (2020*d*); Merad and Martin (2020); Moore and June (2020)

## Materials and methods

For this review, a web-based literature search for all English-language studies or preprints was conducted on PubMed (https://www.ncbi.nlm.nih.gov/pubmed/, last accessed on 8 August 2020), medRxiv and bioRxiv using search terms such as ‘SARS-CoV-2’, ‘2019-nCoV’, ‘novel coronavirus’, ‘COVID-19’, ‘neurology’, ‘neurological disorder’, ‘neurological disease’, ‘neurological complication’, ‘neurological deterioration’, ‘neurological involvement’, ‘central nervous system’, ‘peripheral nervous system’, ‘cerebrospinal fluid’ and ‘brain’, in combination with each other reporting neurological presentations of patients with clinically or laboratory-confirmed SARS-CoV-2 infection. Where available, reviews or brief statements from the national or international neurological societies were taken into account. We have incorporated studies made available online between 1 May and 8 August 2020.

## SARS-CoV-2 and its involvement in neurological diseases

### Meningoencephalitis

At this stage, some case reports have described SARS-CoV-2-associated encephalopathies or meningoencephalitis. It remains, as discussed, unclear whether these diseases result from indirect effects of a systemic pro-inflammatory state, as can be observed in sepsis, or from direct SARS-CoV-2-induced meningeal and neuroglial inflammation. To date, only in a limited number of cases could SARS-CoV-2 be detected in the CNS of patients (Table 3). Moriguchi *et al.* describe a 24-year-old male presenting with headache, fatigue, fever, sore throat, neck stiffness, altered consciousness, pneumonia and new onset generalized seizures. In this case, MRI showed a fluid-attenuated inversion recovery sequence-hyperintensity in the right mesial temporal lobe and hippocampus and a diffusion-weighted magnetic resonance imaging-hyperintensity along the wall of the inferior horn of right lateral ventricle. Furthermore, CSF cell count was mildly elevated to 12/µl and intracranial pressure was >320 mmH_2_O. Interestingly, SARS-CoV-2 RNA was not detected in the nasopharyngeal swab but in the CSF (Moriguchi *et al.*, 2020). The second case report is still unpublished but officially confirmed by the treating medical institution. It describes the case of a 56-year-old patient with viral encephalitis in whose CSF SARS-CoV-2 was identified by gene sequencing (Wu *et al.*, 2020; Xiang *et al.*, 2020; http://xinhuanet.com/english/2020-03/05/c_138846529.htm, accessed on 23 May 2020). The third case was a 41-year-old woman presenting with headache, fever, new onset seizures and signs of meningeal irritation. Cranial CT-scan showed no abnormalities but CSF analysis revealed an increased lymphocytic white cell count of 70/µl and a red cell count of 60/µl without evidence for herpes simplex virus infection. The patient underwent SARS-CoV-2 testing although she had no signs of respiratory discomfort and chest CT did not show any findings suggestive of pneumonia. Both the nasopharyngeal swab test and the CSF sample were positive for SARS-CoV-2 (Duong *et al.*, 2020; Huang *et al.*, 2020*b*). Moreover, Paniz-Mondolfi *et al.* report a SARS-CoV-2-positive Parkinson’s disease patient with initial reduced vigilance, fever and confusion. Although SARS-CoV-2 was not found in the CSF, the virus was detected post-mortem in the frontal lobe by electron microscopy and by reverse transcriptase polymerase chain reaction (RT-PCR; Paniz-Mondolfi *et al.*, 2020). This observation is corroborated by the results of another post-mortem case study in which SARS-CoV-2 RNA was detected in brain tissue from four patients who had died from COVID-19 (Wichmann *et al.*, 2020). In contrast to these studies, Schaller and *et al.* as well as Barton *et al.* did find neither macroscopic nor histological evidence of SARS-CoV-2-related CNS abnormalities in COVID-19 autopsies (Barton *et al.*, 2020; Schaller *et al.*, 2020). On the cellular level, Chu *et al.* detected a modest SARS-CoV-2 replication in the human neuronal U251 cell line (Chu *et al.*, 2020). While this observation suggests that, in principle, CNS cells are susceptible to SARS-CoV-2 infection there are two major caveats. First, the authors did not use primary human CNS cells and second, they observed no substantial cytopathic effect in the cells investigated.

**Table 3:** Cases in which SARS-CoV-2 was detected in the CNS

Authors	Number of patients	Age/gender	Analysis	Manifestation	Imaging
Moriguchi *et al.* (2020)	1	24 years/M	Nasopharyngeal swab SARS-CoV-2 RT-PCR ***negative*** CSF specimen SARS-CoV-2 RT-PCR ***positive***	Pneumonia, headache, fatigue, fever, sore throat, neck stiffness, consciousness disturbance, multiple generalized seizures	Cranial MRI: fluid-attenuated inversion recovery sequence-hyperintensity right mesial temporal lobe and hippocampus. diffusion-weighted magnetic resonance imaging-hyperintensity along the wall of the inferior horn of right lateral ventricle
Xiang *et al.* (2020)	1	56 years/M	CSF specimen SARS-CoV-2 gene sequencing ***positive***	COVID-19, encephalitis	Cranial CT-scan: no abnormal findings
Duong *et al.*, (2020) Huang *et al.* (*2020a*, *b*)	1	41 years/F	Nasopharyngeal swab SARS-CoV-2 RT-PCR ***positive*** CSF specimen SARS-CoV-2 RT-PCR ***positive***	Headache, fever, lethargic, seizure, neck stiffness, photophobia, confusion, hallucinations	Cranial CT-scan: no abnormal findings Serial chest X-ray and chest CT were normal
Paniz-Mondolfi *et al.* (2020)	1	74 years/M	Nasopharyngeal swab SARS-CoV-2 RT-PCR ***negative*** CSF specimen SARS-CoV-2 RT-PCR ***negative*** (post-mortem) Electron microscopy ***positive*** for pleomorphic spherical viral-like 80–110-nm particles frontal lobe brain sections (post-mortem) Minced brain tissue SARS-CoV-2 RT-PCR ***positive***(post-mortem)	COVID-19, fever, confusion, agitation, new onset of atrial fibrillation	Cranial CT-scan: patchy subcortical and periventricular hypodensities unchanged from a scan 6 months earlier
Wichmann *et al.* (2020)	4	n.a.	Minced lung tissue SARS-CoV-2 RT-PCR ***positive***(post-mortem) Minced brain tissue SARS-CoV-2 RT-PCR ***positive***(post-mortem)	COVID-19	n.a.

n.a. = data not given or not available to the authors.

### Cerebrovascular diseases

A potential link between acute cerebrovascular diseases such as ischaemic and haemorrhagic stroke and SARS-CoV-2 infection is controversially discussed. As of now, evidence suggests that this link is CNS unspecific and seems to be rather based on an impact of SARS-CoV-2 on the heart and the peripheral vascular system in the general context of a critical disease. Of note, pre-existing conditions such as arterial hypertension, cardiovascular diseases, diabetes mellitus and smoking predispose patients to develop COVID-19 (Emami *et al.*, 2020) while they are, at the same time risk factors for cerebrovascular disease. It is therefore difficult to disentangle these connections regarding causative versus chance relationship. Nonetheless, a recent study demonstrated that SARS-CoV-2-positive patients who were hospitalized due to stroke showed a higher incidence of fever, delirium and ultimately are at greater risk for poor outcomes than SARS-CoV-2-negative patients (Benussi *et al.*, 2020). Observational studies from Europe and China estimate the proportion of COVID-19 patients with concurrent cerebrovascular disease at 1.3–5.0% (Klok *et al.*, 2020; Li *et al.*, 2020*a*; Lodigiani *et al.*, 2020; Romero-Sánchez *et al.*, 2020). A retrospective observational study by Mao *et al.* reported five cases (2.3%) with ischaemic and one case (0.5%) with haemorrhagic stroke in SARS-CoV-2-positive patients (Mao *et al.*, 2020). These results are supported by several case series describing the occlusion of large arterial vessels in SARS-CoV-2-positive patients (Al Saiegh *et al.*, 2020; Oxley *et al.*, 2020). Pathophysiologically, SARS-CoV-2 could have a direct effect on myocardial and endothelial cells via the ACE2 receptor, which is expressed not only by Type I and Type II alveolar epithelial cells but also by myofibroblasts, vascular endothelial and vascular smooth muscle cells (Hamming *et al.*, 2004). This could result in damage of cell–cell interfaces with subsequent myocardial or vascular cell injury leading to an increased thrombogenicity (Yau *et al.*, 2015). In addition, myofibroblasts activated by SARS-CoV-2 could also interfere with the propagation of electrical signals in the heart resulting in arrhythmia (Quinn *et al.*, 2016). This might, in turn, explain a higher risk for micro- and thrombo-embolic events leading to ischaemic strokes in COVID-19 patients. The same circumstance, i.e. an interrupted cell–cell interface, could underpin reported cases of focal as well as subarachnoid cerebral haemorrhage in COVID-19 patients but, again, this remains to be demonstrated (Heman-Ackah *et al.*, 2020; Hernández-Fernández *et al.*, 2020; Poyiadji *et al.*, 2020; Sharifi-Razavi *et al.*, 2020; Wang *et al.*, 2020*b*). Another possible pathophysiological rationale for the occurrence of cerebrovascular diseases in COVID-19 patients could be the cytokine storm mentioned above. The systemic release of SARS-CoV-2-induced pro-inflammatory cytokines such as IL-1β, IL-6, IL-8 and tumour necrosis factor-α may not only have a negative influence on pre-existing arteriosclerotic diseases [Goldberg *et al.*, 2020, reviewed in Libby *et al.* (2018)]. It could also play an important role in tissue-factor-mediated activation of the coagulation system, *inter alia*, resulting in thrombin generation and the inhibition or the dysfunction of physiological anticoagulant systems (Vary and Kimball, 1992; Levi *et al.*, 1997; Franco *et al.*, 2000; Chen *et al.*, 2020*a*; Fogarty *et al.*, 2020; Harzallah *et al.*, 2020; Helms *et al.*, 2020*b*; Tang *et al.*, 2020; Zhang *et al.*, 2020*b*). Accordingly, this could lead not only to the occurrence of cerebral ischaemia but also to cerebral venous thrombosis in COVID-19 patients as described in several case reports (Garaci *et al.*, 2020; Hemasian and Ansari, 2020; Hughes *et al.*, 2020; Li *et al.*, 2020*a*). Ultimately, this procoagulatory state might activate the fibrinolytic system generating characteristic fibrin degradation products resulting in disseminated intravascular coagulation as it was also observed in COVID-19 patients (Chen *et al.*, 2020*a*; Fogarty *et al.*, 2020; Tang *et al.*, 2020). Another aspect that could explain a hypercoagulable state in COVID-19 patients could be a SARS-CoV-2-induced antiphospholipid syndrome, which by itself is known to affect the CNS causing stroke and cerebral venous thrombosis [reviewed by Nayer and Ortega (2014)]. In a small series of three patients, Zhang *et al.* describe the presence of anticardiolipin immunoglobulin A, anti-β2-glycoprotein I immunoglobulin A and immunoglobulin G antibodies in the serum of patients with COVID-19 who developed cerebral infarctions during their hospitalization (Zhang *et al.*, 2020*b*). Further studies reported 45% up to 88% of COVID-19 patients tested positive for lupus anticoagulant (Harzallah *et al.*, 2020; Helms *et al.*, 2020*b*). However, this might be non-specific since antiphospholipid antibodies and lupus anticoagulants are often detected particularly in elderly patients in the context of infection or related to specific medications [reviewed by Uthman and Gharavi (2002) and Giannakopoulos and Krilis (2013)]. Accordingly, there has been a controversial discussion regarding the significance of these results (Connell *et al.*, 2020; Escher *et al.*, 2020; Tang, 2020; Tang *et al.*, 2020). In addition, Merkler *et al.* report an increased risk of stroke in COVID-19 patients compared to patients who had respiratory tract infection due to another viral pathogen, viral pathogen, even though further studies are needed to confirm this finding (Merkler *et al.*, 2020). Finally, it is worth mentioning that, in general, the fear of contracting COVID-19 has led to a reluctance of patients particularly with mild stroke symptoms to present to emergency departments (Oxley *et al.*, 2020; Siegler *et al.*, 2020). Of note, Zhao *et al.* report a 37.9% decrease in hospital admissions related to stroke during the Chinese epidemic in February 2020 compared to the same period in the previous year. In parallel to that, the absolute number of patients treated by thrombolysis or thrombectomy dropped by 25.5 and 22.7%, respectively. In this regard, not only the patients’ and their families’ fear of contracting SARS-CoV-2 in the hospital may result in a significant delay for timely stroke treatment. The same may apply to insufficient transportation resources during lockdown, COVID-19 screening procedures and local infectious prevention strategies. This could obviously result in potentially poorer outcomes and emphasizes the need for standardized management guidelines in stroke care during the COVID-19 pandemic (Zhao *et al.*, 2020*b*).

### Guillain–Barré-Syndrome

Another conceivable neurological manifestation of SARS-CoV-2 may be Guillain–Barré-Syndrome (GBS) and its variants, which have been reported in a number of COVID-19 patients. GBS is an acute mostly postinfectious immune-mediated disorder affecting nerve roots and peripheral nerves. Clinically, GBS is associated with a rapidly progressive ascending symmetric peripheral paralysis, hypo- or areflexia and can ultimately necessitate mechanical ventilation (Willison *et al.*, 2016). It is usually linked to previous infection with *Campylobacter jejuni*, *Mycoplasma pneumoniae*, Zikavirus, Ebstein-Barr-Virus or other pathogens (Jacobs *et al.*, 1998; Cao-Lormeau *et al.*, 2016; Krauer *et al.*, 2017). Prior to the current pandemic, there have already been reports associating other coronaviruses with different forms of GBS (Kim *et al.*, 2017; Sharma *et al.*, 2019). In the context of the current pandemic, a few case reports have linked GBS and its subforms to prior infection with SARS-CoV-2. The most recent attempt at systematically reviewing reports published before 17 May 2020, found 18 cases (De Sanctis et al., 2020). In nearly all of the cases, GBS symptoms occurred following the clinical manifestation of SARS-CoV-2, i.e. fever and non-productive cough. To our knowledge, there is only one case where symptoms occurred simultaneously (Zhao *et al.*, 2020*a*). Interestingly, in none of the cases SARS-CoV-2 RNA could be detected in the CSF. Moreover, anti-glycolipid antibodies that typically occur in the serum of GBS patients were not detected (Coen *et al.*, 2020; Toscano *et al.*, 2020) although some of the patients were not tested for them (Padroni *et al.*, 2020; Sedaghat and Karimi, 2020; Toscano *et al.*, 2020; Virani *et al.*, 2020). Typical CSF findings such as albuminocytologic dissociation were inconsistent, varying from highly (Coen *et al.*, 2020) to mildly increased (Alberti *et al.*, 2020) to normal protein levels (Toscano *et al.*, 2020). In the reported cases, EMG identified mainly two variants of GBS: motor-sensory demyelinating neuropathy (Alberti *et al.*, 2020; Coen *et al.*, 2020; Toscano *et al.*, 2020; Virani *et al.*, 2020) and motor-sensory axonal neuropathy (Sedaghat and Karimi, 2020; Toscano *et al.*, 2020). Furthermore, three of the reported patients required mechanical ventilation but it is not clear whether respiratory insufficiency resulted from COVID-19 or was part of the natural course of GBS (Toscano *et al.*, 2020). An obvious differential diagnosis would be critical illness polyneuropathy. MRI scans of the brain and spinal cord showed a variety of findings: no pathological signals at all (Sedaghat and Karimi, 2020; Toscano *et al.*, 2020), gadolinium enhancement in the caudal nerve roots or, in one case, bilaterally in the facial nerve (Toscano *et al.*, 2020). In addition, there is one case in which a SARS-CoV-2-positive patient developed Miller–Fisher-Syndrome, a GBS spectrum disease characterized by ataxia, ophthalmoplegia, areflexia and typically antibodies to gangliosides such as ganglioside Q1b. Here, an antibody directed at the ganglioside D1b was identified in the serum and CSF analysis showed albuminocytologic dissociation. However, again SARS-CoV-2 RNA could not be detected in the CSF. The same authors report another case where a SARS-CoV-2-positive patient suffering from diarrhoea developed polyneuritis cranialis. Except for albuminocytologic dissociation, all other laboratory analyses yielded non-specific results (Gutiérrez-Ortiz *et al.*, 2020).

### Acute disseminated encephalomyelitis

Acute disseminated encephalomyelitis (ADEM) is an acute monophasic usually postinfectious, immune-mediated demyelinating disorder of the CNS. As the disease is characterized by multiple white matter lesions in the brain or spinal cord, neurological symptoms can vary significantly (Noorbakhsh *et al.*, 2008). A first case report published in 2003 established a possible link between coronavirus OC43 and ADEM when the virus was detected in the CSF and the nasopharyngeal secretions of a 15-year-old patient (Yeh *et al.*, 2004). As of now, there is only one definite case report of ADEM in an adult female patient who was tested positive for SARS-CoV-2. Analysis of the patient’s CSF yielded no pathological findings, including a negative test for SARS-CoV-2 RNA. MRI revealed multiple white matter lesions in accordance with an ongoing acute inflammatory demyelinating process (Zhang et al., 2020*a*). Another case report by Brun *et al.* describes a 54-year-old woman diagnosed with COVID-19 requiring mechanical ventilation. After sedation was discontinued, the patient presented with prolonged confusion and hemiplegia. MRI revealed homogenous bilateral gadolinium-enhancing brain lesions suggesting ADEM-like demyelination. As a differential diagnosis, the authors discuss small-vessel CNS vasculitis. Once again, CSF RT-PCR for SARS-CoV-2 was negative (Brun *et al.*, 2020). Of note, ADEM as a possible (para-) infectious consequence of COVID-19 is supported by post-mortem neuropathological findings by Reichard *et al.* (2020). The authors describe subcortical scattered clusters of macrophages, a range of associated axonal injury, and a perivascular ADEM-like appearance.

### Acute transverse myelitis

Of note, acute transverse myelitis (ATM) has also been described in the context of SARS-CoV-2. ATM frequently follows infections with various pathogens, particularly viruses. ATM presents with acute paresthaesia, loss of sensation, back pain as well as urinary and bowel incontinence (West *et al.*, 2012). To date, there are two case reports of nasopharyngeal swab SARS-CoV-2-positive patients who developed ATM. Both patients showed the above-described typical clinical symptoms. One of them underwent lumbar puncture. The CSF was, however, negative for SARS-CoV-2 but showed a mononuclear lymphocytosis of 125 cells/μl. MRI of the spinal cord revealed longitudinal signal changes typical of ATM (Sarma and Bilello, 2020). The other patient was diagnosed exclusively based on his clinical symptoms. Neither CSF analysis nor MRI was performed (Zhao et al., 2020*c*).

## Psychiatric and neuropsychiatric presentations

During the last SARS-CoV and MERS coronavirus epidemics, psychiatric and neuropsychiatric symptoms were established as a common feature at times outlasting the infectious disease. Also neuropsychiatric symptoms occurred in caregiving health workers (Sheng *et al.*, 2005; Su *et al.*, 2007; Lancee *et al.*, 2008; Mak *et al.*, 2009; Kim *et al.*, 2018; Lee *et al.*, 2018). Such symptoms included emotional lability, mood disturbances, anxiety, impairment of memory, concentration or attention, sleeping disorders and confusion. In the postinfectious state, SARS-CoV patients presented with a high point-prevalence of anxiety and depression disorders as well as post-traumatic stress disorders at roughly 15–30% even though most of the studies did not include control groups or references from the general population (Rogers *et al.*, 2020). The current evidence for neuropsychiatric complaints in SARS-CoV-2-positive patients is scarce and incomplete regarding the long-term outcomes. In parallel to SARS-CoV and MERS, patients with COVID-19 are more susceptible to develop confusion and delirium (Rogers *et al.*, 2020). On a note of caution, the majority of the mentioned papers have methodological problems (Rogers *et al.*, 2020) so that further studies are required to better delineate SARS-CoV-2-related neuropsychiatric disorders.

## Conclusion

Soon after the COVID-19 pandemic occurred, it became clear that multiple organs other than the lungs are involved. In particular, a number of reports appeared describing a range of neurological disorders putatively associated with it. However, the evidence causally linking SARS-CoV-2 infection to CNS or peripheral nervous system diseases is currently inconclusive. For obvious reasons, most studies were carried out in patients with SARS-CoV-2-associated acute respiratory distress syndrome without matched controls. Patients with such critical conditions are *per se* prone to develop, for instance, stroke, critical illness polyneuropathy, parainfectious GBS and critical illness myopathy (Nauwynck and Huyghens, 1998; Naik-Tolani *et al.*, 1999; Latronico and Bolton, 2011; Walkey *et al.*, 2011; Yuki and Hartung, 2012; Nasr and Rabinstein, 2015). In general, there have only been a limited number of cases in which SARS-CoV-2 was detected in the CNS. Therefore, the nature of a potential impact of SARS-CoV-2 on the nervous system remains presently unclear—all the more so as autopsy results are partly contradictory. In addition, a structured meta-analysis is complicated by the use of different diagnostic tools, small case numbers and the fact that some of the patients suffered from additional, potentially confounding, diseases (Alberti *et al.*, 2020; Sedaghat and Karimi, 2020; Virani *et al.*, 2020). Currently, SARS-CoV-2 not only dominates the scientific discourse but has also an enormous impact on our everyday life as neurologists. Concerns have arisen whether patients with autoimmune nervous system disorders, such as multiple sclerosis or immune neuropathies, should be started or continued on immunomodulatory therapy potentially compromising the capacity to fight off COVID-19 and may modify the risk of developing a severe COVID-19 infection (Amor *et al.*, 2020; Guidon and Amato, 2020; Hartung and Aktas, 2020; Louapre *et al.*, 2020; Parrotta *et al.*, 2020; Rajabally *et al.*, 2020; Sormani, 2020). In general, preliminary evidence suggests that multiple sclerosis patients are not at an increased risk to contract COVID-19 or suffer a more severe form (Louapre *et al.*, 2020; Sormani, 2020). Managing multiple sclerosis patients using a broadened treatment armamentarium creates additional complexity in times of COVID-19 and mandates a personalized approach relying on the unique modes of actions and risks attributable to disease modifying agents (Berger et al., 2020). Fear of infection has a direct impact on patient management and requires the responsible healthcare professional not only to search for possible SARS-CoV-2-related neurological complications or diseases but also to effectively deliver patient care under these challenging circumstances (de Seze and Lebrun-Frenay, 2020; Kim and Grady, 2020; Tarolli *et al.*, 2020). As recently proposed by the World Federation of Neurology, regional, national and international COVID-19 neuro-epidemiological databases are needed to better understand the connection between SARS-CoV-2 and the observed neurological diseases (Román *et al.*, 2020).

## Competing interests

M.F. and V.W. report no disclosures. P.K. is supported by the Stifterverband/Novartisstiftung. M.B. has received institutional support for research, speaking and/or participation in advisory boards for Biogen, Merck, Novartis, Roche, Sanofi Genzyme and Alexion. He is a consulting neurologist for RxMx and research director for the Sydney Neuroimaging Analysis Centre. H.-P.H. has received fees for consulting, speaking and serving on steering committees from Bayer Healthcare, Biogen, GeNeuro, MedImmune, Merck, Novartis, Opexa, Receptos Celgene, Roche, Sanofi Genzyme, CSL Behring, Octapharma and Teva, with approval from the Rector of Heinrich-Heine-University. D.K. received travel grants from GeNeuro and Merck, refund of congress participation fees from GeNeuro, Merck and Servier, consulting fees from Grifols, payment for lectures from Grifols, support for research projects from Teva and was funded by the Deutsche Forschungsgemeinschaft (DFG) while carrying research on human endogenous retroviruses at Cleveland Clinic. The MS Center at the Department of Neurology is supported in part by the Walter and Ilse Rose Foundation and the James and Elisabeth Cloppenburg, Peek, and Cloppenburg Düsseldorf Stiftung.

## Abbreviations

ACE2 =: angiotensin converting enzyme 2;

ADEM =: acute disseminated encephalomyelitis;

ATM =: acute transverse myelitis;

COVID-19 =: coronavirus disease 2019;

GBS =: Guillain–Barré-Syndrome;

IL =: interleukin;

RT-PCR =: reverse transcriptase polymerase chain reaction;

SARS-CoV =: severe acute respiratory syndrome coronavirus;

SARS-CoV-2 =: severe acute respiratory syndrome coronavirus 2
